# Extended antibiotic treatment in salmon farms select multiresistant gut bacteria with a high prevalence of antibiotic resistance genes

**DOI:** 10.1371/journal.pone.0203641

**Published:** 2018-09-11

**Authors:** Sebastián Higuera-Llantén, Felipe Vásquez-Ponce, Beatriz Barrientos-Espinoza, Fernando O. Mardones, Sergio H. Marshall, Jorge Olivares-Pacheco

**Affiliations:** 1 Laboratorio de Genética e Inmunología Molecular, Instituto de Biología, Facultad de Ciencias, Pontificia Universidad Católica de Valparaíso, Campus Curauma, Valparaíso, CP, Chile; 2 Escuela de Medicina Veterinaria, Facultad de Ecología y Recursos Naturales, Universidad Andrés Bello, Republica 252, CP, Santiago, Chile; 3 Millenium Nucleus on Interdisciplinary approach to Antimicrobial Resistance, Lo Barnechea, Santiago, CP, Chile; Nankai University, CHINA

## Abstract

The high use of antibiotics for the treatment of bacterial diseases is one of the main problems in the mass production of animal protein. Salmon farming in Chile is a clear example of the above statement, where more than 5,500 tonnes of antibiotics have been used over the last 10 years. This has caused a great impact both at the production level and on the environment; however, there are still few works in relation to it. In order to demonstrate the impact of the high use of antibiotics on fish gut microbiota, we have selected four salmon farms presenting a similar amount of fish of the Atlantic salmon species (*Salmo salar*), ranging from 4,500 to 6,000 tonnes. All of these farms used treatments with high doses of antibiotics. Thus, 15 healthy fish were selected and euthanised in order to isolate the bacteria resistant to the antibiotics oxytetracycline and florfenicol from the gut microbiota. In total, 47 bacterial isolates resistant to florfenicol and 44 resistant to oxytetracycline were isolated, among which isolates with Minimum Inhibitory Concentrations (MIC) exceeding 2048 μg/mL for florfenicol and 1024 μg/mL for oxytetracycline were found. In addition, another six different antibiotics were tested in order to demonstrate the multiresistance phenomenon. In this regard, six isolates of 91 showed elevated resistance values for the eight tested antibiotics, including florfenicol and oxytetracycline, were found. These bacteria were called “super-resistant” bacteria. This phenotypic resistance was verified at a genotypic level since most isolates showed antibiotic resistance genes (ARGs) to florfenicol and oxytetracycline. Specifically, 77% of antibiotic resistant bacteria showed at least one gene resistant to florfenicol and 89% showed at least one gene resistant to oxytetracycline. In the present study, it was demonstrated that the high use of the antibiotics florfenicol and oxytetracycline has, as a consequence, the selection of multiresistant bacteria in the gut microbiota of farmed fish of the *Salmo salar* species at the seawater stage. Also, the phenotypic resistance of these bacteria can be correlated with the presence of antibiotic resistance genes.

## Introduction

The phenomenon of antibiotic resistance is, according to the General Assembly of the United Nations, a priority topic for human development, being on par with global warming [1]. The number of bacteria resistant to all known antibiotics increases each day, and some have predicted that by 2050, humanity may return to an era without antibiotics [2]. Antibiotics are not limited to medical applications in humans; in fact, notable quantities are used in farm animals and agricultural crops [3,4]. The aims of such usage are to prevent or cure infectious diseases, as well as to promote livestock/crop growth [5]. The issue of antibiotic resistance is further aggravated by climate change, which has accelerated the global food crisis. On this point, estimates indicate that if food production does not improve within the next 40 years, the world will witness a serious global food shortage [6]. Aquaculture is one of the most promising alternatives for efficiently and sustainably increasing the production of animal proteins [7]. Nevertheless, farmed fish are not exempt from antibiotics use. By contrast, antibiotics are routinely administered in aquaculture farms to treat a range of diseases [8]. Antibiotic usage in this industry is largely uncontrolled, and measures must be enacted to prevent consequent harm to human health and the environment [9].

Considering the current and projected situation, antibiotic resistance must be addressed not only from perspectives of human health, but also with considerations to veterinary health and the impact that the use and liberation of antibiotic substances may have on the environment [10]. To this end, the “one-health” concept was developed [9,11] to promote multidisciplinary and multiarea research on antibiotics resistance. One of the principal concerns that arose out of this new investigative paradigm was that of commercial producers of animal proteins [12]. Chile, as the second largest worldwide producer of farmed salmon after Norway [13], has evidenced particular interest in addressing antibiotic resistance in salmon farms.

The Chilean salmon industry is constantly affected by bacterial, parasitic, fungal, and viral infections that cause a series of diseases, many of which can result in the death of millions of fish and, consequently, significant production losses [14]. Without doubt, the pathogen that has mostly plagued the salmon industry over the last 30 years is the facultative intracellular bacterium *Piscirickettsia salmonis*, which is the causative agent of salmonid rickettsial syndrome (SRS) [15]. This bacterium is responsible for more than 80% of fish deaths occuring due to infectious diseases in the three principal fish species farmed in Chile, i.e., Atlantic salmon (*Salmo salar*), coho salmon (*Oncorhynchus kisutch*), and rainbow trout (*O*. *mykiss*) [16]. Although this pathogen is present in other salmonid-producing countries, such as Norway [17], Canada [17,18], Scotland, Ireland [17], and Australia [19], this bacterium is much more aggressive in Chile due to certain genetic traits [15,20]. Although *P*. *salmonis* is the primary pathogen in the Chilean salmon farming, none of the 40 commercially available vaccines are sufficiently effective at protecting against disease [21–24]. Consequently, antibiotics are the primary tool employed in controlling this bacterium, meaning that, over the past 40 years, large quantities of antibiotics have been used by fish farms.

According to a report by the National Fisheries Service (Sernapesca), the salmon industry used more than 5,500 tons of antibiotics between 2007 and 2017, with each ton of produced salmon receiving, on average, 500 g of antibiotics [25]. Antibiotics are mostly used during the fattening stage in marine sites. The two most administered antibiotics in this industry are florfenicol and oxytetracycline, both broad spectrum antibiotcs. In just 2017, 393,9 tons of antibiotics were used, 92,2% of which was florfenicol, and 6,7% of which was oxytetracycline. The remaining 1% corresponded to antibiotics such as erythromycin and amoxycillin [25].

Antibiotics in the aquaculture industry are administered through medicated feed, immersion baths, or, in extreme circumstances, through an intramuscular or intraperitoneal injection [26,27]. Medicated feed is not fully digested by fish and, in many cases, is in fact poorly digested and metabolized, with the consequence being a constant liberation of antibiotic substances into the environment [28]. Without doubt, medicated feed also fundamentally affects the intestinal microbiota of fish [29]. Constant exposure to antibiotic substances leads to the selection of resistant bacteria and an increase in the horizontal transfer of antibiotic-resistance genes (ARGs) [30]. This situation ultimately means that the feces of medicated-feed fish are rich in ARGs.

The primary aim of this study was to characterize the antibiotic-resistant bacteria present in the intestinal microbiota of farmed Atlantic salmon treated with high antibiotic doses. Four salmon farms were sampled, and different antibiotic-resistant bacteria were recorded at each. Specific genetic elements involved in resistance to florfenicol and oxytetracycline were identified, as were integron elements. This paper establishes a clear relationship between the use of antibiotics, the presence of resistant bacteria and the high prevalence of antibiotic resistance genes in the intestinal-microbiota system from the Atlantic Salmon. Additionally, which could be an important source for the dispersion and liberation of ARGs into the environment, with potential impacts for human health.

## Materials and methods

### Sample collection

Four salmon farms located in the Aysén Region, in the Cupquelan Fjord (Northern Patagonia, Chile) were assessed. The farms were chosen for four fundamental characteristics: (i) all the farms contain fish of the *Salmo salar* species in a similar period of the productive cycle; (ii) similar mass of fish (between 4500 and 6000 tons); (iii) all the farms had more than one outbreak of SRS in the productive cycle; and (iv) the four farms had more than one treatment with medicated food at the time of sampling. (Table 1).

**Table 1:** Number of florfenicol (FLO) and oxytetracycline (OTC) oral treatments, sum of the amount of medicated feed with antibiotics and total fish weight in each Atlantic salmon farm at the time that sampling.

Farm	No. treatments and types	Amount of medicated feed with antibiotics (in kg)	Total weight of fish (in tons)
I	3 FCL + 1 OTC	725	5,904
II	4 FCL	807	4,512
III	1 FCL	55	5,262
IV	3 FCL	283	5,578

### Isolation of bacteria from intestinal fish microbiota

In order to obtain the largest number of bacterial isolates, two sources were used: (i) fecal matter; and (ii) the intestines. For this, 15 apparently healthy (*i*.*e*., no clinical signs of SRS) Atlantic salmon were randomly selected from each salmon farm. Fecal matter was obtained by applying perianal stimulation to the fish. Prior to fecal extraction procedure fish were anesthetized with benzocaine (25 μg/mL). The collected fecal samples from three individuals were pooled, stored in a 5 mL saline solution (0.85% NaCl), and kept on ice until laboratory analyses 24 h later. To collect the intestine samples, the animals were euthanized by immersion in a solution of 50 mg/L of benzocaine. The intestines of three fish were also pooled. This study was carried out in accordance with law 20,380 regarding animal welfare, as set out by the Chilean Health Ministry in the use of wild or protected animal species in biomedical research and approved by the National Fisheries Service (SERNAPESCA) and the Pontificia Universidad Católica de Valparaíso Bioethical Committee.

To obtain the bacterial isolates, homogenates were obtained from the pooled fecal matter and intestines. A dilution series was applied, where 10^−4^ and 10^−5^ dilutions (100 μL) were seeded in sextuplet on tryptic soy agar plates (TSA) (Difco, USA). Plates were incubated at 25°C, 30°C, and 37°C for 48 h and at 15°C for 72 h, the aim of which being to assess the widest temperature range possible. TSA medium and incubation temperature have been commonly used to isolate bacteria from fish microbiota [31–33]. To prevent fungus and yeast growth, a 50 μg/mL concentration of cycloheximide was added as an antifungal agent.

### Construction of a bank of bacterial isolates

Colonies grown in TSA medium were classified according to standard patterns: shape, color, texture and shape of the colony-border. Each of the differentiated colonies was seeded in 96-well plates in TSB medium and grown at the temperature at which it was isolated, regardless of whether it came from intestine or fecal material. In order to ensure the purity of the isolates, each of the colonies grown in TSB medium were Gram-characterized and the mixed cultures or cultures contaminated with yeasts were discarded.

### Determinations of minimum inhibitory concentration (MIC)

The MICs for all the bacterial isolates were determined following the agar double-dilution protocol established by the Clinical and Laboratory Standards Institute[34,35]. Briefly, each MIC was estimated by inoculating square plates (120 mm^2^) with the Müller-Hinton medium (Sigma-Aldrich, USA) and increasing concentrations of florfenicol and oxytetracycline (0–2,048 μg/mL), using a 96 pin replicator (Boekel 140500). As a control was used the strain *E*. *coli* K12. Once the MIC of each isolate was calculated, those isolated showing a MIC ≥ 128 μg/mL for florfenicol and ≥ 64 μg/mL for oxytetracycline were considered to be resistant bacteria, according to EUCAST clinical standard [36]. Finally, two banks of resistant bacteria were created–a florfenicol-resistant bank (FB) and an oxytetracycline-resistant bank (OB)

### Determination of the multiresistant phenotype

The cross-resistance potentials of isolates from both banks (*i*.*e*., FB and OB) were measured by estimating MICs to treatment with chloramphenicol (CHL), tetracycline (TET), ciprofloxacin (CYP), erythromycin (ERY), ampicillin (AMP), and kanamycin (KAN). The previously described methodology for establishing MICs was applied, using increasing concentrations of each antibiotic (0–2,048 μg/mL). Resistance levels was defined by the EUCAST values for any antibiotic in *E*. *coli* K12: AMP ≥ 64 μg/mL, CHL ≥ 32 μg/mL, CIP ≥ 4 μg/mL, ERY ≥ 16 μg/mL, KAN 16 μg/mL and TET 32 μg/mL [36]

### Molecular identification of resistant bacteria

To identify the resistant isolates, 16S rRNA gene sequencing analysis was used for taxonomic classification. Genomic DNA extracts were obtained with Chelex-100 (Bio-rad, USA) according to manufacturer instructions. PCR amplification of the 16S gene was carried out using the universal primers 27F (5'-AGAGTTTGATCMTGGCTCAG-3’) and 1492R (5’-GGTTACCTTGTTACGACTT-3’). The amplification conditions were as follows: initial denaturalization at 95°C for 3 min, followed by 35 cycles at 95°C for 45 s, 55°C for 30 s, and 72°C for 1 min, with final extension at 72°C for 5 min. The obtained sequences were analyzed using the BLAST tool [37], with comparisons conducted against sequences available in GenBank (NCBI). Five rounds of amplification and sequenciation were used to verify the sequences. The obtained 16S rRNA sequences for each resistant bacterial isolate were submitted to the GenBank database (S1 Table).

### Characterizing genetic determinants of resistance

The incidences of genetic determinants implicated in resistance to florfenicol and oxytetracycline were evaluated through PCR and sequencing of the most relevant genes described in relation to antibiotic resistance. The partial sequences of the genes (three or four for fish farm) were submitted to the GenBanK database (S2 Table). For florfenicol, these genes included *floR* [38,39], *fexA* [40,41], and *cfr* [42]. For oxytetracycline, these genes included *tetA*, *tetB*, *tetE*, *tetL*, *tetH*, *tetM*, *tet34*, and *tet35* [43]. The primers used for each gene are given in Table 2. All primers were validated with the NCBI primer-Blast tool before their use.

**Table 2:** Primers, amplicon size (base pairs [bp]), annealing temperatures (° C), and references for amplified PCR products.

Primer	Sequence	Annealing	Gene description^PF^	Source	Length
		Temperature			
27 F	5’-AGAGTTTGATCMTGGCTCAG-3’	58°C	16S rDNA	Universal	1460 bp
1492 R	5’-GGTTACCTTGTTACGACTT-3’				
floR F	5'-CCGTCATTCCTCACCTTCAT-3'	58°C	*floR* MFS efflux pump	This study	408 bp
floR R	5'-GACAAGGGAAATGAGCGGTA-3'				
fexA F	5'-TTTCGCTGTTCTTGTGTTCG-3'	56°C	*fexA* MFS efflux pump	This study	358 bp
fexA R	5'-ACCTTGGAAAATCCCCATTC-3'				
cfr F	5'-TGAAGTATAAAGCAGGTTGGGAGTCA-3'	62°C	*cfr* RNAr metiltransferase	This study	746 bp
cfr R	5'-ACCATATAATTGACCACAAGCAGC-3'				
tetL F	5'-TTATCGTTAGCGTGCTGTCATTCC-3'	60°C	*tetL* MFS efflux pump	Miranda et al., (2003)	450 bp
tetL R	5'-TTAAGCAAACTCATTCCAGC-3'				
tetH F	5'-ATACTGCTGATCACCG-3'	56°C	*tetH* MFS efflux pump	Miranda *et al*., (2003)	135 bp
tetH R	5'-TCCCAATAAGCGACGC-3'				
tet34 F	5'-ATGAAAACGAACGCTAATTAACCA-3'	60°C	t*et34* tetracycline resistance gene	Miranda *et al*., (2003)	270 bp
tet34 R	5'-ACATAGAGATCGATGCTAGTACTA-3'				
tet35 F	5'-ATGCGCAAGACCGTCCTAC-3'	60°C	*tet35* MFS efflux pump	Miranda *et al*., (2003)	700 bp
tet35 R	5'-CACACACTAGTAACGGTCGAA-3'				
tetA F	5'-GCGCGATCTGGTTCACTCG -3'	60°C	*tetA* MFS efflux pump	This study	164 bp
tetA R	5'- AGTCGACAGYRGCGCCGGC-3'				
tetB F	5'-TACGTGAATTTATTGCTTCGG-3'	58°C	*tetB* MFS efflux pump	This study	206 bp
tetB R	5'-ATACAGCATCCAAAGCGCAC-3'				
tetE F	5'-GTTATTACGGGAGTTTGTTGG-3'	58°C	*tetE* MFS efflux pump	This study	213 bp
tetE R	5'-AATACAACACCCACACTACGC-3'				
tetM F	5'-GTGGACAAAGGTACAACGAG-3'	58°C	*tetM* tetracycline resistance gene	This study	406 bp
tetM R	5'-CGGTAAAGTTCGTCACACAC-3'				
int1 F	5'-CAGTGGACATAAGCCTGTTC-3'	59°C	Class 1 integrase	This study	160 bp
int1 R	5'-CCCGAGGCATAGACTGTA-3'				

^PF^: Partial fragment of the gene

### Statistical data analysis

Statistical analyses were performed using SPSS 22.0 for Windows (Chicago, IL). The prevalence of antibiotic resistance bacteria and antibiotic resistance genes was compared among isolates from different origins or taxonomic groups using the chi-square test at a significance level of 0.05.

## Results

### Characterization of the bacterial isolates bank

More than 12,000 colonies were obtained in the TSA plates without antibiotics from the four farms sampled. Of these colonies, 28% was obtained at 15°C, while 63% of the isolates came from the growth at 25°C. Finally only 9% of the colonies came from the growth at 30°C. In none of the fish farms were bacteria found that could grow at 37°C. After the classification of the colonies according their pattern of size, shape, color, texture and the shape of the border, and after discarding the mixed cultures or those contaminated with yeast, the number of colonies was considerably reduced: 2,628 coming from the intestine and 2,390 colonies from the feces, showing no differences between this two different sources. The individual analysis per farm showed that 1,248 colonies were obtained from the farm I; while 1,035 are from farm II; 1,472 from farm III; and 1263 from farm IV. The amount and diversity of the patters of size, shape, texture, color and shape of the border from the colonies were similar for the four farms.

### Determinations of MICs for all isolates of the bacterial bank

**Fig 1:**
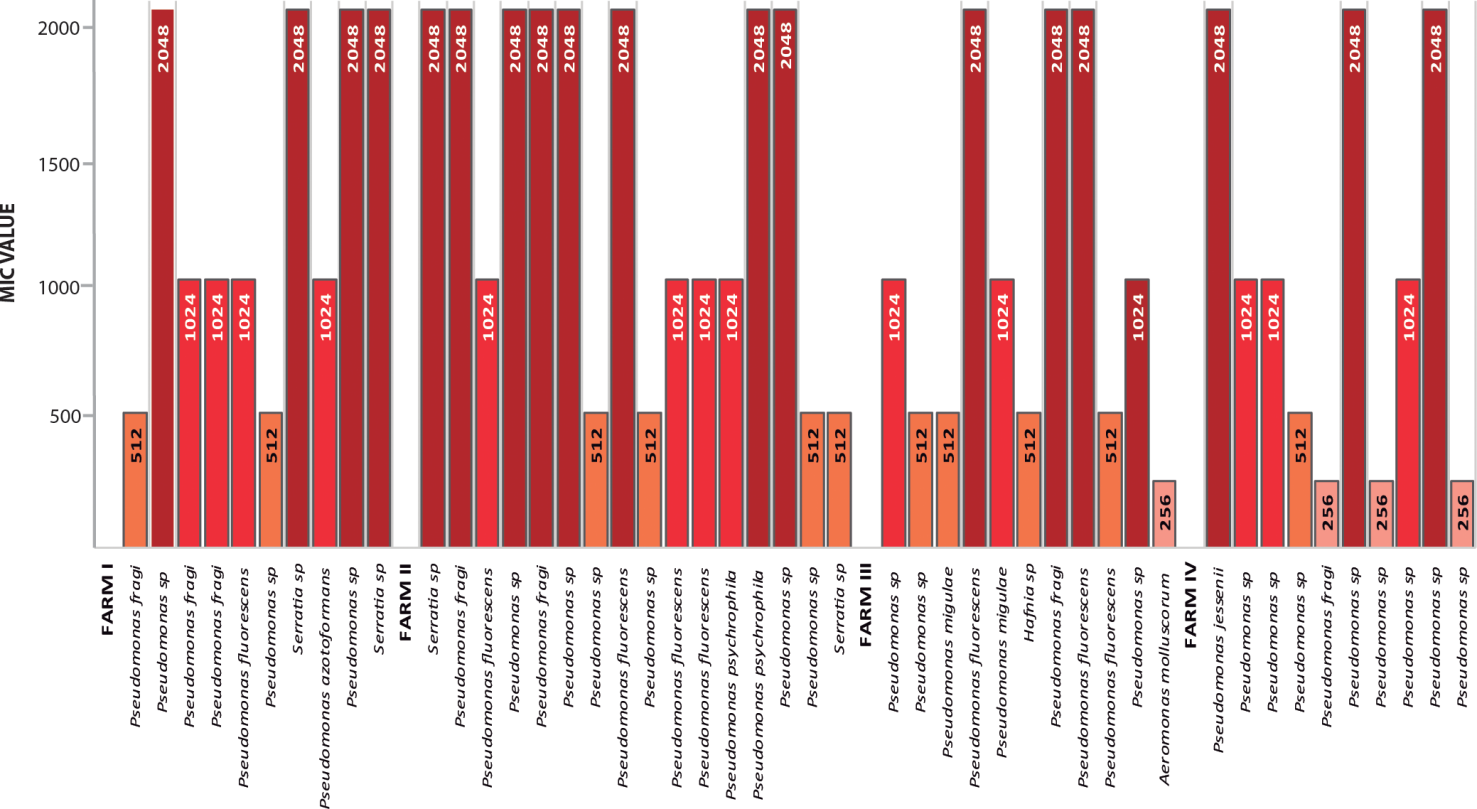
Minimum inhibitory concentrations (MIC) in bacteria resistant to florfenicol. (μg/mL).

**Fig 2:**
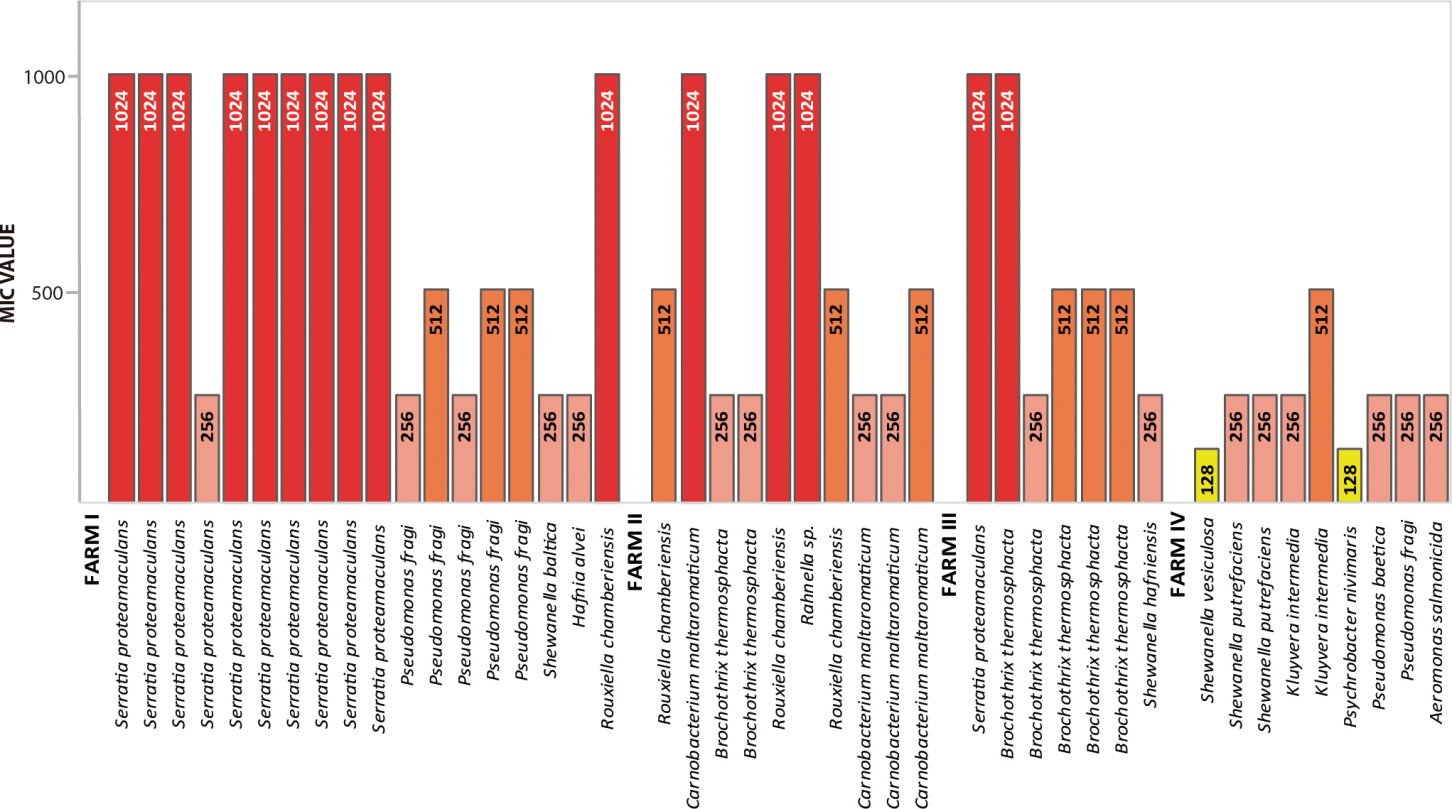
Minimum inhibitory concentrations (MIC) in bacteria resistant to oxytetracycline. (μg/mL).

The MICs to florfenicol or oxytetracycline were determined using microdilutions in Müller-Hinton agar, adding increasing concentrations (0–2,048 μg/mL) of each antibiotic for all isolates of the bacterial bank. Once MICs were determined, those isolates evidencing resistance to ≥64 μg/mL for oxytetracycline or ≥128 μg/mL for florfenicol were classified as resistant. A total of 47 (23 from feces and 24 from intestine) florfenicol-resistant and 44 (21 from feces and 23 from intestine) oxytetracycline-resistant isolates were classified (Figs 1 and 2).

Of the bacteria resistant to florfenicol, 93.6% presented a MIC >256 μg/mL, and 38.2% of these isolates had a MIC > 1,024 μg/mL. Of the bacteria resistant to oxytetracycline, 56.8% presented an MIC >256 μg/mL, and 34.1% of this group had an MIC of 1,024 μg/mL. These findings suggest a high degree of resistance in bacteria from the gut microbiota of fish fed with medicated feed containing these antibiotics. Even at the three farms where medicated food did not contain oxytetracycline, high MICs against this antibiotic were found.

With this data the Antibiotic Resistance Rate (ARR) (resistant bacteria/total culturable bacteria) was calculated, and for the florfenicol resistance samples this rate was estimated in 9.4x10^-3^ and 8.7x10^-4^ for the oxytetracycline resistance samples. In other words, less than 1% of the total isolates were classified as resistant bacteria. If this value is analyzed by farm and by resistant bank, it can be seen that farm I shows a value of 8x10^-3^, while farm II shows a value of 1.2x10^-2^, and farms III and IV show values of 7.4x10^3^ and 7.9x10^-3^ respectively flor florfenicol. In this case the farm II shows an antibiotic resistance rate almost two times higher compared with the others three farms. This results could be explained by the amount of florfenicol treatment used in the farm II. In the case of the OB the values are the following: Farm I: 1.4 x10^-2^; farm II 9.6x10^-3^; farm III 4,7x10^-3^; and farm IV 7.1x10^-3^. In this case it is the farm II that has a value almost two times higher compared to the rest of the farms, which could be explained as it is the only farm where oxytetracycline is used as a treatment in seawater.

### Multiresistant bacterial isolates to florfenicol and oxytetracycline

To estimate the multiresistance capacity against other antibiotics in strains classified as resistant to florfenicol and/or oxytetracycline (*i*.*e*., MIC ≥64 μg/mL for oxytetracycline or ≥128 μg/mL for florfenicol), MIC values were determined against the following antibiotics: chloramphenicol, tetracycline, erythromycin, ampicillin, ciprofloxacin, and kanamycin. Among the FB resistant isolates, 4.3% showed resistance phenotype to the eight assessed antibiotics, and 57.3% showed resistance to at least four antibiotics (Table 3). Different results were found for OB resistant bacteria, with a 8.5% of the bacterial isolates showed resistance to the eight antibiotics. Nevertheless, 100% of OB-classified isolates were resistant to at least four of the tested antibiotics (Table 4). These findings evidence a clear selection tendency for multiresistant bacteria when florfenicol and oxytetracycline are used as treatments.

**Table 3:** Minimum inhibitory concentrations of florfenicol-resistant bacteria to other antibiotic families (μg/mL).

Farm	Species	GenBank Similarity	OTC	CFC	TET	ERY	KAN	CIP	AMP	Isolate	Origin
I	*Pseudomonas fragi*	99%	512	1024	4	128	4	2	128	13H4	F
	*Pseudomonas sp *	99%	512	2048	256	128	8	2	256	13G1	F
	*Pseudomonas fragi*	99%	128	2048	64	1024	8	1	512	7C5	F
	*Pseudomonas fragi*	99%	64	2048	64	128	2	1	128	5B1	I
	*Pseudomonas fluorescens*	99%	512	2048	64	512	8	1	512	7A3	I
	*Pseudomonas sp*	99%	256	2048	256	64	2	1	128	4B2	F
	*Serratia sp*	99%	512	2048	512	512	8	1	>2048	4D1	I
	*Pseudomonas sp*	99%	64	2048	32	128	2	1	128	9A1	I
	*Pseudomonas azotoformans*	99%	512	2048	128	256	2	1	>2048	8C2	F
	*Serratia sp*	99%	512	64	512	256	2	1	512	9A2	I
II	*Serratia sp*	99%	128	512	128	512	4	1	256	1B4	F
	*Pseudomonas fragi*	99%	256	>2048	64	512	4	1	256	1E2	F
	*Pseudomonas fluorescens*	99%	256	>2048	32	512	8	1	256	2B4	F
	*Pseudomonas sp*	99%	32	2048	32	64	4	1	128	1D2	I
	*Pseudomonas fragi*	99%	512	128	32	64	4	1	128	6A4	I
	*Pseudomonas sp*	99%	128	2048	64	512	8	1	128	6C5	F
	*Pseudomonas sp*	99%	8	256	8	1024	32	1	>2048	5B8	I
	*Pseudomonas fluorescens*	99%	64	2048	64	512	4	1	128	6H4	I
	*Pseudomonas sp*	100%	2	256	8	512	32	1	>2048	5C8	I
	*Pseudomonas fluorescens*	99%	128	2048	64	1024	8	1	128	7B8	I
	*Pseudomonas fluorescens*	99%	512	16	256	1	2	1	16	6D3	F
	*Pseudomonas psychrophila*	99%	64	16	32	1	2	1	16	6A5	I
	*Pseudomonas psychrophila*	99%	512	>2048	512	>2048	2048	1024	>2048	4G7	I
	*Pseudomonas sp*	99%	2	2048	16	64	4	1	128	6H3	F
	*Pseudomonas sp*	100%	64	256	128	512	128	1	128	4D7	F
III	*Pseudomonas sp *	98%	4	512	4	1024	32	1	256	1A8	I
	*Pseudomonas sp*	100%	4	512	4	1024	32	1	256	1C7	F
	*Pseudomonas migulae*	99%	4	128	4	512	2	1	256	3C4	F
	*Pseudomonas fluorescens*	99%	512	512	128	512	8	1	>2048	4A11	F
	*Pseudomonas migulae*	99%	2	64	256	1024	8	1	>2048	7F11	I
	*Hafnia sp*	99%	256	128	256	2048	8	1	128	6B1	F
	*Pseudomonas fragi*	99%	64	2048	32	128	2	1	64	8B12	I
	*Pseudomonas fluorescens*	99%	64	64	32	128	2	1	128	8F12	F
	*Pseudomonas fluorescens*	99%	2	256	4	512	32	1	>2048	11H8	F
	*Pseudomonas sp*	99%	2	64	4	1024	32	1	>2048	9F10	I
	*Aeromonas molluscorum*	99%	2	64	4	64	8	1	>2048	9E11	I
IV	*Pseudomonas jessenii*	99%	16	1024	16	512	4	1	256	1G10	I
	*Pseudomonas sp *	99%	32	2048	32	128	4	2	128	1H11	F
	*Pseudomonas sp *	99%	32	>2048	32	256	0	1	256	1F11	F
	*Pseudomonas sp *	99%	4	128	4	512	4	1	256	3A8	F
	*Pseudomonas fragi*	99%	64	2048	64	512	8	1	128	5B4	I
	*Pseudomonas sp*	99%	512	>2048	512	>2048	>2048	2048	>2048	4C3	F
	*Pseudomonas sp*	99%	2	64	4	512	2	1	>2048	10G1	F
	*Pseudomonas sp*	99%	32	64	8	32	1	1	64	8A7	I
	*Pseudomonas sp*	99%	16	256	4	16	1	1	256	9B4	I
	*Pseudomonas sp*	99%	2	64	4	1024	2	1	>2048	9C6	F

AMP (ampiciline), CHL (chloramphenicol), CIP (ciprofloxacin), ERY (erythromycin), OTC (Oxytetracycline), KAN (kanamycin), TET (tetracycline). As a control was used the strain *E*. *coli* K12. Resistance levels was defined by the EUCAST values for any antibiotic. AMP ≥64 μg/ml, CHL ≥32 μg/ml, CIP ≥4, ERY ≥16 μg/ml, KAN ≥16 μg/ml, TET ≥32 μg/ml. Origin: F = Fecal matter; I: Intestine

**Table 4:** Minimum inhibitory concentrations of oxytetracycline -resistant bacteria to other antibiotic families (μg/mL).

Farm	Species	GenBank Similarity	FLO	CFC	TET	ERY	KAN	CIP	AMP	Isolate	Origin
I	*Serratia proteamaculans*	96%	2048	2048	1024	512	128	8	2	P151C9	I
	*Serratia proteamaculans*	96%	2048	2048	1024	512	128	8	4	P151E2	I
	*Serratia proteamaculans*	98%	512	2048	1024	512	128	8	2	25P1E12	F
	*Serratia proteamaculans*	99%	512	512	512	512	128	8	2	30P1XC9	I
	*Serratia proteamaculans*	99%	512	512	512	1024	1024	512	2048	30P1XC8	I
	*Serratia proteamaculans*	99%	512	512	256	128	512	8	1024	30P1XD3	I
	*Serratia proteamaculans*	99%	512	512	512	256	512	8	2	30PF8	F
	*Serratia proteamaculans*	99%	512	512	256	128	512	8	32	30PF10	I
	*Serratia proteamaculans*	99%	512	512	256	128	512	8	32	30PF11	I
	*Serratia proteamaculans*	98%	512	512	512	128	512	8	2	30P1XE10	I
	*Pseudomonas fragi*	99%	512	512	256	128	512	8	2	30P1XB10	F
	*Pseudomonas fragi*	100%	1024	512	256	64	32	8	32	25P1H2	I
	*Pseudomonas fragi*	100%	512	512	256	32	512	8	2	P30F12	I
	*Pseudomonas fragi*	100%	1024	1024	512	512	1024	512	32	25P1D4	I
	*Pseudomonas fragi*	98%	512	512	256	128	512	8	2	30P1XE12	F
	*Shewanella baltica*	96%	16	16	64	128	32	8	2	30P1XB8	F
	*Hafnia alvei*	99%	1024	1024	32	128	1024	8	2	25P3D1	I
	*Rouxiella chamberiensis*	99%	512	512	256	128	32	8	32	30PXG6	I
II	*Rouxiella chamberiensis*	99%	2048	1024	256	32	8	2	128	154F5	F
	*Carnobacterium maltaromaticum*	100%	1024	1024	512	1024	512	2048	>2048	25P2A9	F
	*Brochothrix thermosphacta*	97%	512	256	64	512	8	2	128	P30XA10	F
	*Brochothrix thermosphacta*	97%	512	256	128	32	8	2	128	P30XB10	I
	*Rouxiella chamberiensis*	100%	512	256	128	32	8	2	256	30PXB9	F
	*Rahnella sp*.	99%	256	256	128	32	8	2	256	30PXG7	I
	*Rouxiella chamberiensis*	99%	256	256	64	512	8	2	128	P30XB3	F
	*Carnobacterium maltaromaticum*	100%	512	256	64	512	8	2	128	P30XD4	F
	*Carnobacterium maltaromaticum*	100%	256	256	64	512	8	2	128	P30XD5	I
	*Carnobacterium maltaromaticum*	100%	512	256	128	32	512	16	128	P30XG8	F
III	*Serratia proteamaculans*	98%	1024	512	512	128	8	32	256	25P1F12	F
	*Brochothrix thermosphacta*	100%	512	32	128	32	8	2	64	25P3C5	I
	*Brochothrix thermosphacta*	100%	512	256	128	512	8	2	64	30P1XG3	F
	*Brochothrix thermosphacta*	100%	256	256	64	32	8	2	64	P30C3	I
	*Brochothrix thermosphacta*	100%	512	256	64	32	8	2	64	P30C4	I
	*Brochothrix thermosphacta*	100%	512	256	64	32	8	2	64	P30C5	I
	*Shewanella hafniensis*	99%	256	256	64	512	8	2	64	30PF3	F
IV	*Shewnella vesiculosa*	99%	256	64	16	64	8	2	64	15P2G11	F
	*Shewanella putrefaciens*	99%	64	32	128	64	8	2	64	25P3F1	I
	*Shewanella putrefaciens*	99%	2048	256	128	128	8	4	128	15P3A4	F
	*Kluyvera intermedia*	94%	2048	1024	256	128	8	2	128	15P2D11	F
	*Kluyvera intermedia*	99%	2048	1024	256	128	8	2	128	15P2G8	I
	*Psychrobacter nivimaris*	99%	256	256	16	64	8	4	64	15P2H7	F
	*Pseudomonas baetica*	99%	256	32	32	1024	8	2	512	25P2F9	I
	*Pseudomonas fragi*	98%	256	512	256	128	8	2048	64	25P2F4	F
	*Aeromonas salmonicida*	95%	512	32	32	64	8	2	64	30PB8	F

Antibiotic used, AMP (ampiciline), CHL (chloramphenicol), CIP (ciprofloxacin), ERY (erythromycin), FLO (florfenicol), KAN (kanamycin), TET (tetracycline). As a control was used the strain *E*. *coli* K12. Resistance levels was defined by the EUCAST values for any antibiotic. AMP ≥64 μg/ml, CHL ≥32 μg/ml, CIP ≥4, ERY ≥16 μg/ml, KAN ≥16 μg/ml, TET ≥32 μg/ml. Origin: F = Fecal matter; I: Intestine

Particularly interesting results were found for resistance tests against ciprofloxacin and kanamycin, because only two of the florfenicol-resistant isolates showed resistance for ciprofloxacin (Table 3). But in the case of the OB a 54.5% of the bacterial isolates showed resistance to ciprofloxacin (Table 4). Similar results were found when testing against the antibiotic kanamycin–only nine florfenicol-resistant bacteria evidenced a resistance phenotype against kanamycin (Table 3). In turn, 45.4% of those bacteria resistants to oxytetracycline were also resistant to kanamycin, and nearly all of these bacteria originated for farm I (Table 4).

It is important to mention that only six bacterial isolates, two from the FB and four from the OB showed resistance to the eight antibiotics evaluated. The two isolates of the FB correspond to members of the *Pseudomonas* genus, while of the OB two of them were classified as member of the *Serratia* genus and two of the *Carnobacterium* genus. Among the six identified multiresistant bacteria, a *Pseudomonas* sp. from the FB is particularly concerning since this bacterium presented a MIC ≥ 2,048 μg/mL to six of the eight evaluated antibiotics. Considering this finding, the assessed *Psudomonas* sp. isolate was termed “super-resistant” and is currently being sequenced for continued research.

### Genetic determinants involved in antimicrobial resistance

After an exhaustive analysis of phenotypic resistance, a search was conducted for genetic elements directly involved in resistance to florfenicol and oxytetracycline. Integron elements were also evaluated since these elements can collect antibiotic-resistance genes (ARGs), thus facilitating expression [44]. These elements can not only incorporate and actively express ARGs, because if they are incorporated in mobile genetics elements like plasmids, they can be easily transferred also, increasing considerably the dispersion of those genes [45,46]. As expected, FB resistant bacteria showed a high incidence of *floR* and *fexA*, genes that code for efflux pumps from the major facilitator superfamily group MFS, that expulse a significant number of molecules, including antibiotics, thereby conferring resistance to florfenicol. Of the bacterial isolates with the FB, 70.2% carried *floR*, 46.8% carried *fexA*, and 34% evidenced both genes. Only 17% did not carry either *floR* or *fexA* (Table 5). Regarding bacteria from the OB, none carried *floR*. However, 52.2% did present *fexA* (Table 6). These results support that determinants of florfenicol resistance are widely distributed across the gut microbiota of farmed fish as a result of high antibiotics administration.

**Table 5:** Antibiotic resistance genes amplified by PCR and sequenced in bacteria resistant to florfenicol.

Farm	Species	*floR*	*fexA*	*tet34*	*tet35*	*tetA*	*tetB*	*tetE*	*tetH*	*tetL*	*tetM*	*int1*	Isolate	Origin
I	*Pseudomonas fragi*	-	+	-	-	-	-	-	-	-	+	+	13H4	F
	*Pseudomonas sp *	-	+	-	-	+	-	-	-	-	-	+	13G1	F
	*Pseudomonas fragi*	+	+	-	-	-	-	-	+	+	-	+	7C5	F
	*Pseudomonas fragi*	+	+	-	-	-	-	+	+	+	-	+	5B1	I
	*Pseudomonas fluorescens*	+	+	-	-	-	-	-	+	+	-	+	7A3	I
	*Pseudomonas sp*	+	-	-	-	+	-	+	+	-	-	-	4B2	F
	*Serratia sp*	+	+	-	-	+	-	-	-	-	-	+	4D1	I
	*Pseudomonas sp*	+	-	-	-	-	-	-	+	-	-	+	9A1	I
	*Pseudomonas azotoformans*	+	+	-	-	-	-	-	+	-	-	+	8C2	F
	*Serratia sp*	+	+	-	-	-	-	+	+	-	-	+	9A2	I
II	*Serratia sp*	+	-	-	-	-	+	-	+	-	-	+	1B4	F
	*Pseudomonas fragi*	-	-	-	-	-	+	-	+	-	-	+	1E2	F
	*Pseudomonas fluorescens*	+	+	-	-	-	-	-	+	+	+	+	2B4	F
	*Pseudomonas sp *	+	-	-	-	-	-	-	+	-	-	+	1D2	I
	*Pseudomonas fragi*	+	-	-	-	-	-	+	-	-	-	+	6A4	I
	*Pseudomonas sp*	+	+	-	-	-	-	-	+	+	-	+	6C5	F
	*Pseudomonas sp*	+	-	-	-	-	-	-	-	-	-	+	5B8	I
	*Pseudomonas fluorescens*	+	+	-	-	-	-	-	-	-	-	+	6H4	I
	*Pseudomonas sp*	-	-	-	-	-	-	-	-	-	-	+	5C8	I
	*Pseudomonas fluorescens*	+	+	-	-	-	-	-	+	-	-	+	7B8	I
	*Pseudomonas fluorescens*	+	-	-	-	-	-	-	-	-	+	+	6D3	F
	*Pseudomonas psychrophila*	+	+	-	-	-	-	-	-	+	-	+	6A5	I
	*Pseudomonas psychrophila*	+	-	-	-	-	-	-	-	-	-	+	4G7	I
	*Pseudomonas sp*	-	+	-	-	-	-	-	-	+	-	+	6H3	F
	*Pseudomonas sp*	+	-	-	-	-	-	-	+	-	+	+	4D7	F
III	*Pseudomonas sp *	+	-	-	-	-	-	-	-	-	-	+	1A8	I
	*Pseudomonas sp*	-	-	-	-	-	-	-	-	-	-	+	1C7	F
	*Pseudomonas migulae*	-	-	-	-	-	-	+	-	-	-	+	3C4	F
	*Pseudomonas fluorescens*	+	+	-	-	-	-	+	+	-	+	+	4A11	F
	*Pseudomonas migulae*	+	+	-	-	+	-	-	-	-	-	+	7F11	I
	*Hafnia sp*	+	-	-	-	+	-	+	-	-	-	-	6B1	F
	*Pseudomonas fragi*	-	-	-	-	-	-	-	+	-	-	+	8B12	I
	*Pseudomonas fluorescens*	+	+	-	-	-	-	-	+	-	-	+	8F12	F
	*Pseudomonas fluorescens*	-	-	-	-	-	-	-	-	+	-	+	11H8	F
	*Pseudomonas sp*	+	+	-	-	-	-	-	-	-	-	+	9F10	I
	*Aeromonas molluscorum*	ND	+	-	-	-	-	-	-	-	-	+	9E11	I
IV	*Pseudomonas jessenii*	+	-	-	-	-	-	-	+	-	-	+	1G10	I
	*Pseudomonas sp*	+	-	-	-	+	-	+	+	-	-	+	1H11	F
	*Pseudomonas sp*	+	-	-	-	+	-	-	+	-	-	+	1F11	F
	*Pseudomonas sp*	-	-	-	-	-	-	+	-	-	-	+	3A8	F
	*Pseudomonas fragi*	+	+	-	-	-	-	-	+	+	-	+	5B4	I
	*Pseudomonas sp*	-	+	-	-	-	-	-	+	-	+	+	4C3	F
	*Pseudomonas sp*	-	-	-	-	-	-	-	-	-	-	+	10G1	F
	*Pseudomonas sp*	+	-	-	-	-	-	-	-	+	-	+	8A7	I
	*Pseudomonas sp*	ND	-	-	-	+	-	+	+	+	-	-	9B4	I
	*Pseudomonas sp*	+	+	-	-	-	-	-	-	-	-	+	9C6	F

(+) Presence of the gene; (-) Absence of the gene; (ND) Not Determined.

**Table 6:** Antibiotic resistance genes amplified by PCR and sequenced in bacteria resistant to oxytetracycline.

Farm	Species	*floR*	*fexA*	*tet34*	*tet35*	*tetA*	*tetB*	*tetE*	*tetH*	*tetL*	*tetM*	*Int1*	Isolate	Origin
I	*Serratia proteamaculans*	-	-	-	+	+	+	-	-	+	+	+	P151C9	I
	*Serratia proteamaculans*	-	-	-	-	-	+	-	-	+	+	+	P151E2	I
	*Serratia proteamaculans*	-	-	-	+	-	+	+	-	-	+	+	25P1E12	F
	*Serratia proteamaculans*	-	-	-	-	-	+	+	-	+	+	+	30P1XC9	I
	*Serratia proteamaculans*	-	-	-	-	-	+	+	-	+	-	+	30P1XC8	I
	*Serratia proteamaculans*	-	+	-	-	+	+	+	-	+	-	+	30P1XD3	I
	*Serratia proteamaculans*	-	+	-	-	-	+	+	-	+	+	+	30PF8	F
	*Serratia proteamaculans*	-	+	-	-	-	+	+	-	+	+	+	30PF10	I
	*Serratia proteamaculans*	-	+	-	-	-	+	+	-	+	-	+	30PF11	I
	*Serratia proteamaculans*	-	-	-	-	-	+	+	-	+	-	+	30P1XE10	I
	*Pseudomonas fragi*	-	+	-	+	+	+	-	+	-	+	+	30P1XB10	F
	*Pseudomonas fragi*	-	-	-	+	+	-	-	+	+	+	+	25P1H2	I
	*Pseudomonas fragi*	-	+	+	-	+	-	-	-	-	+	+	P30F12	I
	*Pseudomonas fragi*	-	-	-	+	+	-	-	+	-	-	+	25P1D4	I
	*Pseudomonas fragi*	-	+	-	-	+	-	-	-	-	+	+	30P1XE12	F
	*Shewanella baltica*	-	+	-	-	-	+	-	-	+	+	+	30P1XB8	F
	*Hafnia alvei*	-	-	-	-	-	-	-	-	-	+	+	25P3D1	I
	*Rouxiella chamberiensis*	-	+	-	-	-	+	-	+	+	+	+	30PXG6	I
II	*Rouxiella chamberiensis*	-	-	-	-	+	-	+	-	-	+	+	154F5	F
	*Carnobacterium maltaromaticum*	-	-	-	-	-	-	-	-	+	+	+	25P2A9	F
	*Brochothrix thermosphacta*	-	+	-	-	-	-	+	-	+	+	+	P30XA10	F
	*Brochothrix thermosphacta*	-	+	-	-	-	-	-	-	+	+	+	P30XB10	I
	*Rouxiella chamberiensis*	-	+	-	-	-	-	-	+	+	+	+	30PXB9	F
	*Rahnella sp*.	-	+	+	-	-	-	-	+	+	+	+	30PXF7	I
	*Rouxiella chamberiensis*	-	+	-	-	-	-	-	+	+	+	+	P30XB3	F
	*Carnobacterium maltaromaticum*	-	+	-	-	+	-	+	-	+	+	+	P30XD4	F
	*Carnobacterium maltaromaticum*	-	+	-	-	-	+	-	-	+	+	+	P30XD5	I
	*Carnobacterium maltaromaticum*	-	+	-	-	-	-	+	-	+	-	+	P30XG8	F
III	*Serratia proteamaculans*	-	-	-	-	+	+	+	-	+	+	+	25P1F12	F
	*Brochothrix thermosphacta*	-	+	-	-	+	-	-	-	+	+	+	25P3C5	I
	*Brochothrix thermosphacta*	-	ND	-	-	+	-	-	-	+	+	+	30P1XG3	F
	*Brochothrix thermosphacta*	-	+	-	-	-	-	-	-	+	+	+	P30C3	I
	*Brochothrix thermosphacta*	-	+	-	-	-	-	-	+	+	+	+	P30C4	I
	*Brochothrix thermosphacta*	-	ND	-	-	-	-	+	-	+	+	+	P30C5	I
	*Shewanella hafniensis*	-	+	-	+	-	+	+	+	+	+	+	30PF3	F
IV	*Shewnella vesiculosa*	-	+	-	-	-	+	-	-	-	-	+	15P2G11	F
	*Shewanella putrefaciens*	-	+	-	-	-	+	+	-	-	-	+	25P3F1	I
	*Shewanella putrefaciens*	-	-	-	+	-	+	-	-	-	+	+	15P3A4	F
	*Kluyvera intermedia*	-	+	-	-	-	-	+	-	-	-	+	15P2D11	F
	*Kluyvera intermedia*	-	-	-	+	*-*	-	+	-	-	+	+	15P2G8	I
	*Psychrobacter nivimaris*	-	ND	-	-	+	-	-	-	+	-	+	15P2H7	F
	*Pseudomonas baetica*	-	-	-	+	+	+	+	+	-	+	+	25P2F9	I
	*Pseudomonas fragi*	-	-	-	-	-	-	+	-	-	-	+	25P2F4	F
	*Aeromonas salmonicida*	-	ND	-	-	+	+	+	-	+	-	+	30PB8	F

(+) Presence of the gene; (-) Absence of the gene; (ND) Not Determined.

After assessing the presence of florfenicol-resistance genes among the FB and OB isolates, analyses were conducted for genes connected to oxytetracycline resistance. Eight resistance genes were included in evaluations, all of which confer resistance to the tetracycline drug family. These apply named *tet* genes included *tetA*, *tetB*, *tetE*, *tetH*, *tetL*, *tet34*, and *tet 35*, all of which were previously described in sediments associated with salmon farms [43], but not with intestinal microbiota of the farmed fish. The presence of *tetM* was also included considering prior reports of being a widely dispersed and prevalent resistance mechanism against tetracyclines [47,48]. For OB resistance bacteria, 100% of isoaltes presented at least one of the evaluated *tet* genes, the more prevalent of which was, not surprisingly, *tetM* (72.7%). Following in prevalence was *tetL* (68.1%). Among this group of bacterial isolates, 4.5% of the bacterial isolates carried six *tet* genes, 9,1% five and 15.9% four. In other words, 29.5% of oxytetracycline-resistant bacteria carried at least four different resistance genes to this antibiotic (Table 6). Regarding those bacteria included in the FB, 78.7% of isolates had at least one of the evaluate *tet* genes, 48.9% of which were carries of *tetH* (*i*.*e*., the most abundant in florfenicol-resistant bacteria; Table 5). Likewise, an estimated 12.8% of the FB isolates carried at least three of the evaluated *tet* genes. The presence of specific ARGs against florfenicol and oxytetracycline corroborate the phenotypic resistances observed for each of the resistant isolates. Further research is needed regarding those resistant isolates for which no ARGs were detected. To understand these results, it is necessary to take into account the limitations of detection using the PCR technique, since only eight out of 28 *tet* genes were evaluated [49], the most frequently described in aquaculture [50]. The same happens with the genes of resistance to phenicoles since only those that are detected more frequently at environmental level were evaluated [38,51]. Therefore a metagenomic analysis would be very useful to detect all the possible genes involved in the resistance against these antibiotics.

Once resistance elements to the most widely used antibiotics were determined, a search for integron-like elements was conducted. The presence of type I integrase was established through PCR analyses and posterior sequencing. In the case of OB isolates, 100% were positive for type I integrase, while 96% of FB isolates presented type I integrase (Tables 5 and 6). These results suggest that this type of element might be widely distributed among resistant bacteria residing within the gut microbiota of fish. These are highly dangerous elements that play a fundamental role in the distribution and phenotypic expression of ARGs. A dedicated study on the detected integrons is currently underway in our lab.

### Taxonomic differences among bacteria resistant to florfenicol and oxytetracycline

In contrast to previously published reports, the present study simultaneously provides information related to the phenomena of resistance to florfenicol and oxytetracycline, with the different tested isolates having been directly obtained from the intestinal microbiota of farmed salmon exposed to high concentrations of antibiotics. Of note, the conducted analyses detected differences in species richness for resistant bacteria. More specifically, the FB isolates evidenced a high predominance of *Pseudomonas* species, a phenomenon that was found across the sampled farming centers and among all the evaluated temperatures. To a lesser degree, members of the Gammaprotebacteria class were also isolated, including *Aeromonas*, *Serratia*, and *Hafnia* species. Regarding OB isolates, a high incidence of Gammaprotebacteria were also observed, as represented by the genera *Serratia*, *Hafnia*, *Rouxiella*, *Rahnella*, *Kluyvera*, *Shewanella*, *psychrobacter*, *Aeromonas*, and *Pseudomonas*. Furthermore, Gram-positive bacteria were represented by the Firmicutes phylum, notable among which were *Carnobacterium* and *Brochothrix*, among others.

## Discussion

Antibiotic use in the production of animal proteins is a practice that should be controlled on a global scale, specifically since the application of these medicines for veterinary ends many times lacks the degree of strict control imposed on human medicines. One of the primary methods for administering antibiotics to industrially farmed animals is medicated feed [52–54]. This delivery method is the most popular among farmed fish due to the notable technical difficulties associated with administering antibiotics through other means, such as injection [55,56]. The ingestion of medicated feed implicates that the intestinal microbiota of these animals is strongly affected. Antibiotics substantially change the bacterial diversity of gut microbiota in farmed animals [53], which, often, can result in functional and growth problems [57]. Another effect caused by the high use of the medicated food on the intestinal microbiota is that the feces could contain resistant bacteria, which could be easily dispersed in the environment [58,59]. In this work it was possible to demonstrate that the feces contain multiresistant bacteria, which are constantly being released into the environment. Additionally, it was observed that the diversity of resistant isolates is similar to that found in the intestine, reaffirming in this way that eventually any resistant bacteria selected in the intestine carrying ARGs could be disseminated through the feces.

Various studies have extensively characterized the gut-microbiota composition of Atlantic salmon, reporting total dominance by the Proteobacteria phylum. More specific still, *Pseudomonas* species of the Gammaproteobacteria class are the most represented [29,60]. These prior descriptions were supported by the present study, with most of the resistant isolates being of the *Pseudomonas* genus. Prior research has similarly characterized the effect that oxytetracycline use has on the microbiota composition of Atlantic salmon. For example, Navarrete *et al*. (2008) [29] exposed Atlantic salmon to high oxytetracycline concentrations under experimental conditions (*i*.*e*., not in the field) and found a notable enrichment of *Aeromonas salmonicida*, a bacterial pathogen. In the present study, only one *Aeromonas* isolate was found in the OB group, thus corroborating the possible selection of this bacterium under high oxytetracycline concentrations.

A fundamental finding of the present study is that oxytetracycline-resistant isolates were found to be more diverse than those resistant to florfenicol. This could have direct implications on treatments and the strategies applied to the use of these antibiotics. Since the early report of *P*. *salmonis* infection by the end of the 80s, oxytetracycline and quinolones have been used to tackle the infection. Also, oxytetracycline is the most widely used antibiotic during freshwater rearing, with florfenicol use nearly null during this stage [25]. Consequently, the large majority of farmed fish with a freshwater stage have been subjected to oxytetracycline treatments [25]. This could, in turn, mean that the gut microbiota of these fish is already rich in bacteria resistant to oxytetracycline. Little research has reported on substantial changes to the microbiota of freshwater- versus seawater-stage salmon. However, some authors speculate that a large part of the microbiota would be conserved between systems [61]. In contrast to freshwater farming, florfenicol is the most widely used antibiotic in seawater farming. Supporting this claim, all of the currently assessed fish farms had applied three to four oral applications of florfenicol, and only farm I had used an additional treatment of oxytetracycline at the time of sampling. This might explain the lesser diversity of bacteria resistant to florfenicol; *i*.*e*., the constant florfenicol treatments may have selected and maintained only those bacteria with the most efficient mechanisms against this antibiotic. This is the perfect scenario for *Pseudomonas* members, the genomes of which are equipped with multiple resistance elements that make these bacteria competitive in environments saturated with antibiotics [62,63].

In addition to the well-known antibiotic resistance capacity of *Pseudomonas*, the present study detected most of the resistance genes included in assessments, and the majority of these genes, excepting *tetM*, were drug efflux pumps. These findings corroborate that these genetic elements are a principal trait of *Pseudomonas* bacteria [64]. Considering the phenotypic versatility of *Pseudomonas*, it was unsurprising that the majority of the obtained florfenicol-resistant isolates were of this genus. Regarding oxytetracycline-resistant isolates, the detected genera, as with *Pseudomonas*, are highly ubiquitous in different systems. For example, *Hafnia alvei* has been isolated from the intestinal microbiota of various organisms, including pigs, cows, and fish [65,66]. This bacterium is an important contaminant of packaged food [67], meaning that *H*. *alvei* is an ideal resistant-isolate candidate in any system. These traits can also be found in species of *Shewanella* [68,69], *Serratia* [70,71], *Carnobacterium* [72], *Brochotrhix* [73,74], and *Kluyvera* [75,76]. All of these bacteria can colonize multiple environments, and the ability to acquire resistance in any given environment represents a significant risk for human health. Only *Aeromonas molluscorum* [77,78] and *Psychrobacter nivimaris* [79] are exclusive to the aquatic environment, which translates into a highly probable exchange of ARGs between bacteria directly related to the human system and environmental bacteria from the gut microbiota of fish.

Tetracyclines are commonly used in veterinary medicine around the world, and, specifically, oxytetracycline is the most widely used antibiotic during the freshwater stage of salmon growth in Chile [25]. Origin records for the Atlantic salmon sampled in the present study were not obtained, but it is very likely that the sampled fish were exposed to high doses of oxytetracycline during the freshwater stage. This would explain the high instance of oxytetracycline-resistant bacteria, even when oxytetracycline was not applied during the seawater stage. Similarly, the high prevalence of *tet* genes found for the FB and OB could be explained by the high frequency at which these genes localize in mobile genetic elements, such as plasmids or transposons [49]. The genes *tetA*, *tetB*, *tetE*, *tetL*, *tetH*, *tet34*, and *tet35* have been recorded in the sediments and water column of Chilean salmon farms [43,80]. An interisting case is the gene *tetM*, this globally distributed gene presents a clinically confirmed resistance mechanism to tetracyclines [81,82]. One trait of this gene is that it is frequently associated with transposons and plasmids in human pathogenic bacteria [83,84]; this could be a clear sign of the impacts this gene is having from the clinical to the environmental spheres. Consequently, antibiotics used in industrially farmed animals could substantially contribute to the maintenance and dispersion of ARGs in natural environments.

As with the *tet* genes, the presence of *floR*, a drug efflux pump of the major facilitator superfamily (MFS), has previously been reported in bacteria collected from marine sediments associated with salmon farming in Chile [39]. This gene has even been detected in a plasmid that also transported *tet* and *qnr* genes, the latter of which are implicated in resistance to quinolones [85]. However, the present study is the first to report on the existence of *floR* in the gut microbiota of salmon. Furthermore, this investigation is the first to find and report on *fexA*, an efflux pump of the major facilitator superfamily also, in association with Chilean salmon farming, with no previously published study having detected this gene in relation to the Chilean aquaculture industry. This fenicol-resistance gene was first described in the bacterium *Staphylococcus lentus* [40], and has since almost been exclusively described in members of the *Staphylococcus* genus [41,42,51,86–88], both for humans and animals, but never in the marine environment. Apart from the *Staphylococcus* genus, *fexA* has been described in members of the *Enterococcus* genus, specifically in association with industrially farmed terrestrial animals [89,90]. No *Staphylococcus* or *Enterococcus* bacteria were found among any of the obtained resistant isolates, but the presented findings do support the ability of *fexA* to disperse across a wide variety of both Gram-positive and–negative bacteria.

One curious outcome of the conducted study was that none of the oxytetracycline-resistant bacteria presented *floR*, but, by contrast, 52,2% did present *fexA*. As mentioned, the most widely used antibiotic used in Chilean salmon farming is oxytetracycline, and there is a nearly null use of florfenicol in the freshwater stage. Prior reports have found *fexA* in plasmids containing a great number of resistance genes to other antibiotics [87]. Therefore, this gene has probably been selected during the freshwater stage in plasmids that also carry tetracylcin-resistance genes. This co-selection is likely since the water used in the freshwater rearing stage is collected from the lakes and rivers most exposed to human activities, including livestock farms, and, as stated, *fexA* has almost exclusively been isolated from industrially farmed terrestrial animals. This is another compelling case for ARGs primarily originating from human activities and not directly from salmon farming.

In contrast to findings from sediments related to intense salmon farming in Chile [91], the present study found a high incidence of class I integrons in resistant isolates from both the FB and OB databases. In fact, 100% of OB isolates and 96% of FB isolates were positive for type I integrase. These genetic elements are the greatest contributors to the evolution and dispersion of ARGs [92]. Integrons are true genetic platforms that acquire exogenous genes through mobile genetic cassettes, having the ability to collect up to hundreds of genes in the same unit [93]. Nevertheless, these elements did not originate in bacterial pathogens, nor is their primary function to collect antibiotic-resistance genes. Integrons frequently appear in *Betaproteobacteria* native to soils, freshwater, and saltwater. Genetic cassettes are incorporated alongside elements that are highly varied and much more diverse than ARGs [94]. Integrons are very successful and ubiquitous in natural environments, and it is therefore unsurprising that the majority of the isolated OB and FB bacteria would present these elements. Concern arises when these elements have a clinical origin and carry ARGs for most of the known antibiotics [95]. Integrons carrying ARGs against quinolones (*qnrA* and *qnrB*), trimethoprims (*drfA12*), and aminoglycosides (*aad2*) have recently been described in the Chilean farming system, which is in addition to some clinical *Escherichia coli* also carrying these plasmid-transferred integrons [96]. This appears to be credible proof that there is an interaction between the clinical environment and that of salmon farming. However, a number of questions remain–What is the origin of these integrons? Are they from aquaculture-associated bacteria? Or have contributions from the clinical environment contaminated bacteria from salmon farming? These questions need to be answered. No integron from either the OB or FB bacteria presented the traits described by Tomova *et al*. (2018) [90].

Another important aspect to consider in this work is the Antibiotic Resistance Rate (ARR). Overall, it was found that less than 1% of the total bacterial isolates were resistant to oxytetracycline or florfenicol (0.94% for florfenicol and 0.87% for oxytetracycline). Although there are few works in which this rate is addressed at an environmental level [97,98], values close to 1% can be considered high [97–100]. In these works it can be seen that when the concentration of antibiotics in the medium increases, the value of this range increases too. This phenomenon is corroborated in the present work since the ARR in the FB for farm II exceeds 1%, almost twice as much as that presented by the other farms and it is precisely the farm II that the major number of treatments with florfenicol presents, four. The same effect is observed in the OB in farm I where ARR again exceeds 1% and the farm I is the only one that uses oxytetracycline as a treatment in its seawater stage.

The information collected in the present study clearly indicate that using large amounts of antibiotics to treat industrially farmed animals has the consequence of selecting for multiresistant bacteria in the intestinal microbiota, which then have undeniable advantages when liberated into the environment through the feces. While the obtained results are somewhat biased towards “culturable” bacteria, metagenomics data are currently being analyzed, the results of which will reveal all of the bacteria that did not grow in a TSA medium. This information will provide a complete picture for the behaviors of the bacterial populations interacting in this complex system. Meanwhile, the currently reported data do show that the intestinal microbiota, as a system “semi-isolated” from the environment yet in direct contact with applied antibiotics, is the perfect place for interactions to occur between bacteria from different environments, including from the human clinical context. Therefore, the gut microbiota of farmed salmon could also serve as the perfect reservoir for ARGs, the dispersion of which would be fundamentally related to feces. The release of feces with resistant bacteria and ARGs is creating an environment constantly rich in resistance elements, creating an ideal situation for genetic exchanges to occur between different bacterial populations, whether in the water column or in marine sediments. These populations are precisely where fish pathogens can be found, thus notably increasing the risk that these bacteria will acquire resistance elements. If this occurs, no effective treatments will exist for the control of these pathogens. Fortunately, a number of aquaculture producers are beginning to understand the gravity of the situation. Measures aimed at decreasing antibiotic usage are increasingly being adopted, with expectations that by 2020, there will be at least a 50% decrease in the use of these substances.

## Conclusion

In this study, bacterial isolates from fish gut microbiota of the *Salmo salar* species, showing high resistance levels to antibiotics oxytetracycline and florfenicol, were characterised. The feces, represent important vehicles of dispersion of resistant bacteria and ARGs. The analyses showed that the high use of these antibiotics selects bacteria that are multiresistant to a wide range of antibiotics. In turn, these bacteria show a high prevalence of antibiotic resistance genes, thus, verifying the phenotypic resistance with the mechanism present in the bacteria to cope with these substances. Furthermore, almost 100% of these (antibiotic) resistant isolates showed class 1 integrons, dangerous elements involved in the resistance phenomenon due to their capacity of dispersion.

## Supporting information

S1 Table Genbank access numbers for 16S rRNA genes amplified from different bacterial isolates. (DOCX)

S2 Table Genbank access numbers for the partial sequence of the antibiotic resistance genes (ARGs) amplified by PCR. (DOCX)
