# Supplementary material for: Extended antibiotic treatment in salmon farms select multiresistant gut bacteria with a high prevalence of antibiotic resistance genes
Source: PLoS One. 2018 Sep 11;13(9):e0203641. doi: 10.1371/journal.pone.0203641 (PMC6133359; doi:10.1371/journal.pone.0203641)
Supplement: S1 Table — (DOCX) [file pone.0203641.s001.docx]

**Supporting Information 1**

**S1 Table.** Genbank access numbers for 16S rRNA genes amplified from different bacterial isolates.

| **Source** | **Bank** | **Genus/species** | **Isolate** | **Accesion number** |  | **Source** | **Bank** | **Genus/species** | **Isolate** | **Accesion number** |
| --- | --- | --- | --- | --- | --- | --- | --- | --- | --- | --- |
| Farm I | FCL | *Pseudomonas fragi* | 13H4 | MH000209 |  | Farm II | OXT | *Rouxiella chamberiensis* | 154F5 | KY963430 |
|  | FCL | *Pseudomonas sp* | 13G1 | KY940314 |  |  | OXT | *Carnobacterium maltaromaticum* | 25P2A9 | KY963433 |
|  | FCL | *Pseudomonas fragi* | 7C5 | MH000210 |  |  | OXT | *Brochothrix thermosphacta* | P30XA10 | KY963444 |
|  | FCL | *Pseudomonas fragi* | 5B1 | MH000208 |  |  | OXT | *Brochothrix thermosphacta* | P30XB10 | KY963445 |
|  | FCL | *Pseudomonas fluorescens* | 7A3 | KY940317 |  |  | OXT | *Rouxiella chamberiensis* | 30PXB9 | KY963446 |
|  | FCL | *Pseudomonas sp* | 4B2 | KY940318 |  |  | OXT | *Rahnella sp.* | 30PXG7 | KY963448 |
|  | FCL | *Serratia sp* | 4D1 | KY940320 |  |  | OXT | *Rouxiella chamberiensis* | P30XB3 | KY963449 |
|  | FCL | *Pseudomonas sp* | 9A1 | MH000377 |  |  | OXT | *Carnobacterium maltaromaticum* | P30XD4 | KY963451 |
|  | FCL | *Pseudomonas azotoformans* | 8C2 | KY940319 |  |  | OXT | *Carnobacterium maltaromaticum* | P30XD5 | KY963452 |
|  | FCL | *Serratia sp* | 9A2 | KY940321 |  |  | OXT | *Carnobacterium maltaromaticum* | P30XG8 | KY963453 |
|  | OXT | *Serratia proteamaculans* | P151C9 | KY963460 |  | Farm III | FCL | *Pseudomonas sp* | 1A8 | KY940348 |
|  | OXT | *Serratia proteamaculans* | P151E2 | KY963461 |  |  | FCL | *Pseudomonas sp* | 1C7 | KY940350 |
|  | OXT | *Serratia proteamaculans* | 25P1E12 | KY963467 |  |  | FCL | *Pseudomonas migulae* | 3C4 | KY940346 |
|  | OXT | *Serratia proteamaculans* | 30P1XC9 | KY963468 |  |  | FCL | *Pseudomonas fluorescens* | 4A11 | KY940342 |
|  | OXT | *Serratia proteamaculans* | 30P1XC8 | KY963469 |  |  | FCL | *Pseudomonas migulae* | 7F11 | KY940345 |
|  | OXT | *Serratia proteamaculans* | 30P1XD3 | KY963470 |  |  | FCL | *Hafnia sp* | 6B1 | KY940351 |
|  | OXT | *Serratia proteamaculans* | 30PF8 | KY963471 |  |  | FCL | *Pseudomonas fragi* | 8B12 | KY940323 |
|  | OXT | *Serratia proteamaculans* | 30PF10 | KY963472 |  |  | FCL | *Pseudomonas fluorescens* | 8F12 | KY940343 |
|  | OXT | *Serratia proteamaculans* | 30PF11 | KY963472 |  |  | FCL | *Pseudomonas fluorescens* | 11H8 | KY940344 |
|  | OXT | *Serratia proteamaculans* | 30P1XE10 | MH000379 |  |  | FCL | *Pseudomonas sp* | 9F10 | KY940347 |
|  | OXT | *Pseudomonas fragi* | 30P1XB10 | KY963473 |  |  | FCL | *Aeromonas molluscorum* | 7E11 | KY940322 |
|  | OXT | *Pseudomonas fragi* | 25P1H2 | KY963474 |  |  | OXT | *Serratia proteamaculans* | 25P1F12 | KY963432 |
|  | OXT | *Pseudomonas fragi* | P30F12 | KY963475 |  |  | OXT | *Brochothrix thermosphacta* | 25P3C5 | KY963437 |
|  | OXT | *Pseudomonas fragi* | 25P1D4 | KY963476 |  |  | OXT | *Brochothrix thermosphacta* | 30P1XG3 | KY963441 |
|  | OXT | *Pseudomonas fragi* | 30P1XE12 | KY963477 |  |  | OXT | *Brochothrix thermosphacta* | P30C3 | KY963442 |
|  | OXT | *Shewanella baltica* | 30P1XB8 | KY963478 |  |  | OXT | *Brochothrix thermosphacta* | P30C4 | MH000378 |
|  | OXT | *Hafnia alvei* | 25P3D1 | KY963479 |  |  | OXT | *Brochothrix thermosphacta* | P30C5 | KY963443 |
|  | OXT | *Rouxiella chamberiensis* | 30PXG6 | KY963480 |  |  | OXT | *Shewanella hafniensis* | 30PF3 | KY963440 |
| Farm II | FCL | *Serratia sp* | 1B4 | KY940338 |  | Farm IV | FCL | *Pseudomonas jessenii* | 1G10 | KY940353 |
|  | FCL | *Pseudomonas fragi* | 1,00E+02 | KY940326 |  |  | FCL | *Pseudomonas sp* | 1H11 | MH000215 |
|  | FCL | *Pseudomonas fluorescens* | 2B4 | KY940328 |  |  | FCL | *Pseudomonas sp* | 1F11 | KY940356 |
|  | FCL | *Pseudomonas sp* | 1D2 | MH000211 |  |  | FCL | *Pseudomonas sp* | 3A8 | KY940358 |
|  | FCL | *Pseudomonas fragi* | 6A4 | MH000212 |  |  | FCL | *Pseudomonas fragi* | 5B4 | KY940325 |
|  | FCL | *Pseudomonas sp* | 6C5 | KY940312 |  |  | FCL | *Pseudomonas sp* | 4C3 | MH000216 |
|  | FCL | *Pseudomonas sp* | 5B8 | MH000213 |  |  | FCL | *Pseudomonas sp* | 10G1 | KY940359 |
|  | FCL | *Pseudomonas fluorescens* | 6H4 | KY940327 |  |  | FCL | *Pseudomonas sp* | 8A7 | KY940357 |
|  | FCL | *Pseudomonas sp* | 5C8 | KY940335 |  |  | FCL | *Pseudomonas sp* | 9B4 | KY940355 |
|  | FCL | *Pseudomonas fluorescens* | 7B8 | KY940331 |  |  | FCL | *Pseudomonas sp* | 9C6 | MH000217 |
|  | FCL | *Pseudomonas fluorescens* | 6D3 | KY940337 |  |  | OXT | *Shewnella vesiculosa* | 15P2G11 | KY963425 |
|  | FCL | *Pseudomonas psychrophila* | 6A5 | KY940332 |  |  | OXT | *Shewanella putrefaciens* | 25P3F1 | KY963435 |
|  | FCL | *Pseudomonas psychrophila* | 4G7 | KY940333 |  |  | OXT | *Shewanella putrefaciens* | 15P3A4 | KY963426 |
|  | FCL | *Pseudomonas sp* | 6H3 | MH000214 |  |  | OXT | *Kluyvera intermedia* | 15P2D11 | KY963427 |
|  | FCL | *Pseudomonas sp* | 4D7 | KY940336 |  |  | OXT | *Kluyvera intermedia* | 15P2G8 | KY963428 |
|  | FCL | *Serratia sp* | 6D3 | KY940330 |  |  | OXT | *Psychrobacter nivimaris* | 15P2H7 | KY963429 |
|  |  |  |  |  |  |  | OXT | *Pseudomonas baetica* | 25P2F9 | KY963434 |
|  |  |  |  |  |  |  | OXT | *Pseudomonas fragi* | 25P2F4 | KY963436 |
|  |  |  |  |  |  |  | OXT | *Aeromonas salmonicida* | 30PB8 | KY963439 |
