# Supplementary material for: Extended antibiotic treatment in salmon farms select multiresistant gut bacteria with a high prevalence of antibiotic resistance genes
Source: PLoS One. 2018 Sep 11;13(9):e0203641. doi: 10.1371/journal.pone.0203641 (PMC6133359; doi:10.1371/journal.pone.0203641)
Supplement: S2 Table — (DOCX) [file pone.0203641.s002.docx]

**Supporting Information 2**

**S2 Table.** Genbank access numbers for the partial sequence of the antibiotic resistance genes (ARGs) amplified by PCR

| **Bank** | **Source** | **Genus** | **Isolate** | **Gene** | **Accession number** |
| --- | --- | --- | --- | --- | --- |
| FCL | Farm I | *Pseudomonas fragi* | 5B1 | fexA, partial sequence | MH747496 |
| FCL | Farm I | *Pseudomonas poae* | A2E5 | fexA, partial sequence | MH747497 |
| FCL | Farm II | *Pseudomonas psychrophila* | 6A5 | fexA, partial sequence | MH747498 |
| FCL | Farm II | *Pseudomonas fragi* | 6C5 | fexA, partial sequence | MH747499 |
| FCL | Farm II | *Listeria monocytogenes* | 12A3 | fexA, partial sequence | MH747495 |
| FCL | Farm II | *Hafnia alvei* | 12D5 | fexA, complete sequence | MH747503 |
| FCL | Farm III | *Staphylococcus equorum* | 12C5 | fexA, partial sequence | MH747500 |
| FCL | Farm III | *Pseudomonas fluorescens* | 4A11 | fexA, partial sequence | MH747501 |
| FCL | Farm III | *Enterococcus faecium* | 12C8 | fexA, partial sequence | MH747502 |
| FCL | Farm III | *Aeromonas molluscorum* | 9E11 | fexA, partial sequence | MH747494 |
| FCL | Farm I | *Serratia sp* | 4D1 | floR, partial sequence | MH747483 |
| FCL | Farm I | *Pseudomonas poae* | A2E5 | floR, partial sequence | MH747487 |
| FCL | Farm I | *Pseudomonas fragi* | 5B1 | floR, partial sequence | MH747490 |
| FCL | Farm II | *Listeria monocytogenes* | 12A3 | floR, partial sequence | MH747491 |
| FCL | Farm II | *Pseudomonas fluorescens* | 6H4 | floR, partial sequence | MH747486 |
| FCL | Farm II | *Pseudomonas psychrophila* | 6A5 | floR, partial sequence | MH747488 |
| FCL | Farm II | *Pseudomonas fragi* | 6A4 | floR, complete sequence | MH747493 |
| FCL | Farm III | *Hafnia sp* | 6B1 | floR, partial sequence | MH747489 |
| FCL | Farm III | *Enterococcus faecium* | 12C8 | floR, partial sequence | MH747485 |
| FCL | Farm III | *Staphylococcus equorum* | 12C5 | floR, partial sequence | MH747492 |
| FCL | Farm IV | *Pseudomonas jessenii* | 1G10 | floR, partial sequence | MH747484 |
| OXT | Farm III | *Brochothrix thermosphacta* | 25P3C5 | tetA, partial sequence | MH747508 |
| OXT | Farm I | *Serratia sp* | P151C9 | tetH, partial sequence | MH747505 |
| OXT | Farm III | *Brochothrix sp* | P30C4 | tetH, partial sequence | MH747507 |
| OXT | Farm IV | *Kluyvera sp* | 12P2G8 | tetH, partial sequence | MH747506 |
| OXT | Farm IV | *Shewanella* | 15P3A4 | tetH, partial sequence | MH747504 |
| OXT | Farm I | *Pseudomonas sp* | 25P1H2 | tetL, partial sequence | MH747509 |
| OXT | Farm II | *Carnobacterium sp* | P30XD5 | tetL, partial sequence | MH747514 |
| OXT | Farm III | *Brochothrix sp* | 25P3C5 | tetL, partial sequence | MH747510 |
| OXT | Farm III | *Rouxiella sp* | P30XC3 | tetL, partial sequence | MH747511 |
| OXT | Farm III | *Brochothrix sp* | P30C4 | tetL, partial sequence | MH747513 |
| OXT | Farm IV | *Shewanella sp* | 15P3A4 | tetL, partial sequence | MH747512 |
| OXT | Farm I | *Serratia sp* | P151C9 | tetM, partial sequence | MH747516 |
| OXT | Farm IV | *Kluyvera sp* | 12P2G8 | tetM,, partial sequence | MH747515 |
